# Supplementary material for: Beta traveling waves in monkey frontal and parietal areas encode recent reward history
Source: Nat Commun. 2023 Sep 5;14:5428. doi: 10.1038/s41467-023-41125-9 (PMC10480436; doi:10.1038/s41467-023-41125-9)
Supplement: Supplementary file 1 — Supplementary Information [file 41467_2023_41125_MOESM1_ESM.pdf]

Supplemental Material

Beta traveling waves in the monkey frontoparietal network predict recent reward memory

Erfan Zabeh[1,2], Nicholas C Foley[2], Joshua Jacobs[1,3*], Jacqueline. Gottlieb[2,4,5*]

Department of Biomedical Engineering, Columbia University[1],
Mortimer B. Zuckerman Mind Brain Behavior Institute[2],
Department of Neurological Surgery, Columbia University[3],
Department of Neuroscience, Columbia University[4],
Kavli Institute for Brain Science, Columbia University

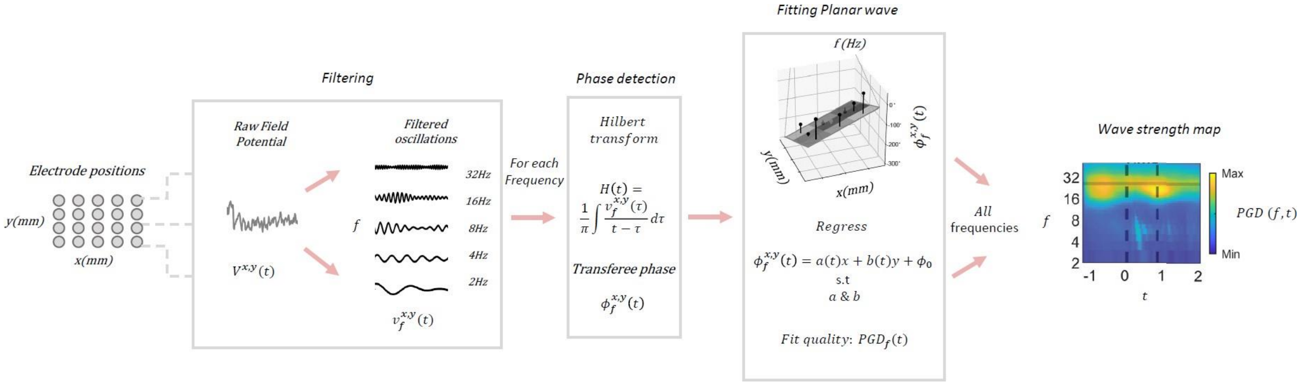

Fig. S1 PGD calculation. *Flowchart illustrating the analysis paradigm for obtaining PGD maps from the raw data. For each recording electrode in position (x,y), the raw LFP signal $V x,y(t)$ was separated into its oscillatory components $v\ x,y(t)$ within a frequency range (f) from 2 to 50 Hz. For each frequency, the phase of the oscillation was extracted using the Hilbert transform. The phases across all the electrodes were then fit as a function of space. The quality of the fit was computed as the PGD, which thus indicated the strength of the TW – i.e., the extent to which phase changed in consistent (linear) manner across the array. PGD were calculated for each frequency and time point and displayed as time-frequency maps.*

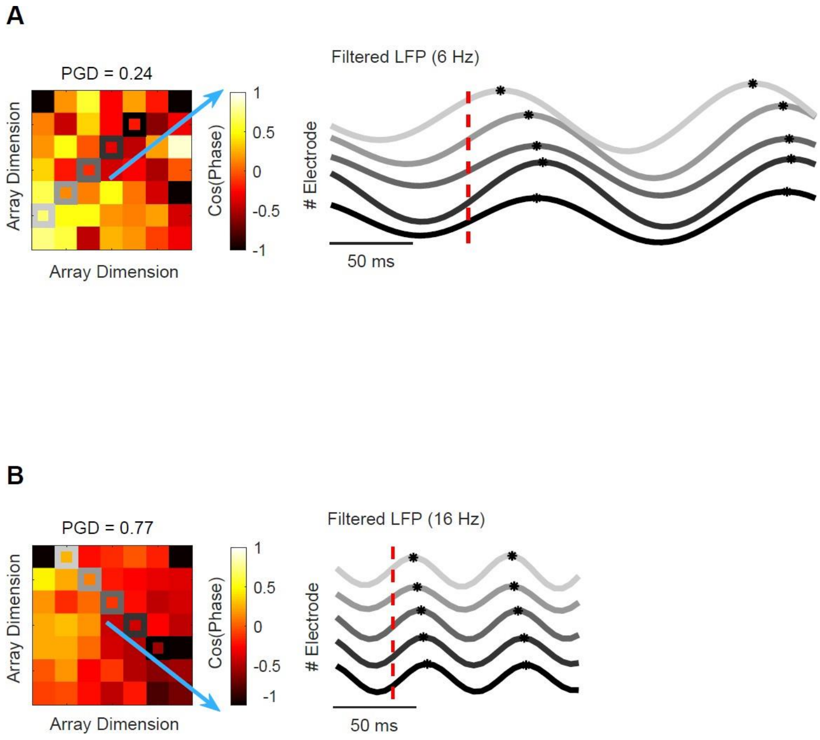

Fig. S2. Example of high and low frequency TW. (A) Low frequency TW. *Representative trial from monkey Mj's PPC array showing a low-frequency oscillation (filtered at 6Hz during the pre-cue epoch). The left phase map illustrates the spatial distribution of phases across the recording array at a single time point (indicated by the dashed red line in the right panel). The blue arrow represents the overall direction of phase propagation. The right panel shows the filtered LFPs at adjacent electrodes (shown in light to dark gray in the phase map). Black stars indicate oscillation peaks showing a systematic phase shift across adjacent electrodes. The red line is the timepoint shown in the phase map.* ***(B) High frequency oscillation as TW.*** *A different trial from the same array, in the same format as in* ***A****, showing a high-frequency oscillation (filtered at 16 Hz).*

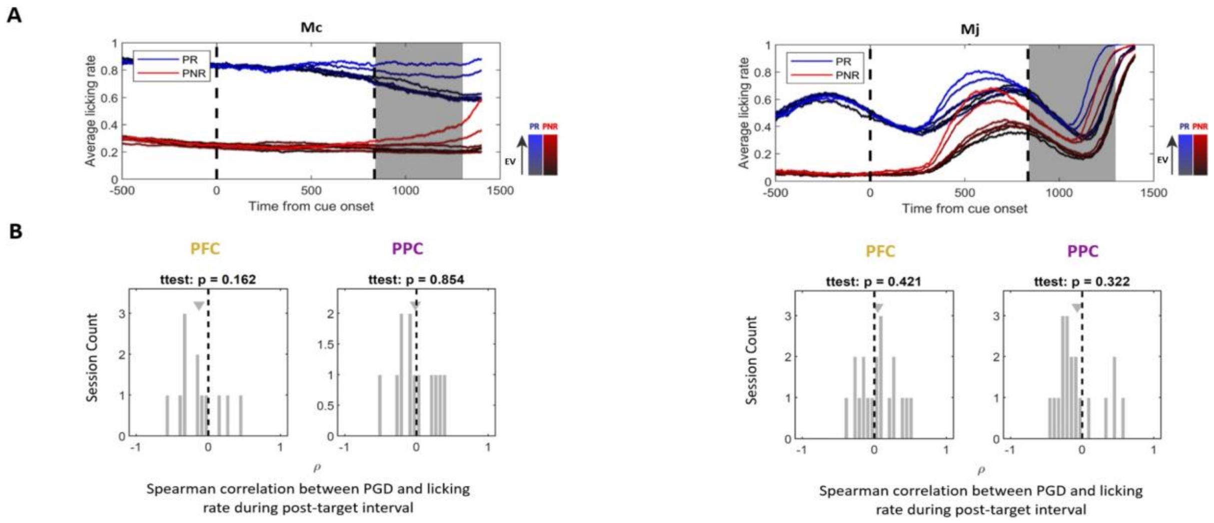

Fig. S3. Alternative analysis of relation between PGD and licking over time. (A) Licking rate dynamics. *Top panels represent the temporal dynamics of licking rate, separately computed for PR and PNR trials with different expected values (blacker coloring indicate lower EV). Licking rates peak shortly before the anticipated time of reward delivery.* ***(B) Analysis of the correlation between licking and wave strength around the time of reward delivery.*** *The average licking rate within the shaded interval (500ms post-target interval) was z-scored based on PR condition and then binned by the expected value on each trial. Horizontal axis indicates the spearman correlation between wave strength (measured by PGD) and the z-scored licking rate in the same interval, for each session. Plot indicates a histogram of the spearman correlation coefficient. None of the distributions significantly differ from zero (two sided t-test, p's>0.1), indicating that wave strength fluctuations are independent of licking rate.*

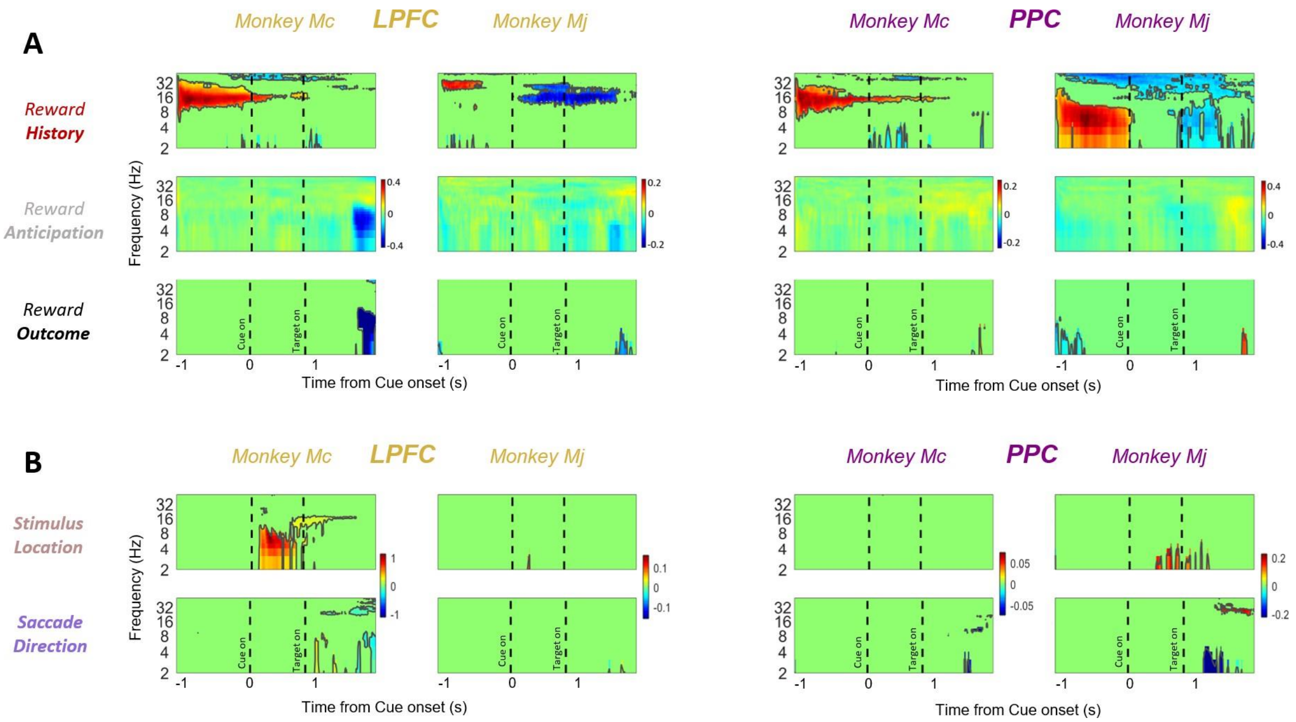

Fig. S4. TW strength does not correlate with other reward or spatial variables (A) *Each panel shows the result of a linear model relating TW strength with different reward parameters, across time and frequency map. Conventions as in* ***Fig. 4D****. Top: Prior Reward (Reward History), identical to* ***Fig. 4D****, Middle: Expected Value of Reward (Reward Anticipation), bottom: Current Reward (Reward Outcome). The dashed lines represent the times of cue onset (0s) and target onset (0.835 s).* ***(B) Model coefficients relating TW strength to visuomotor behaviors.*** *Although some modulations are present at low frequencies, they are inconsistent, occurring at different timepoints and frequencies compared to the prior reward effects, indicating that they are distinct phenomena.*

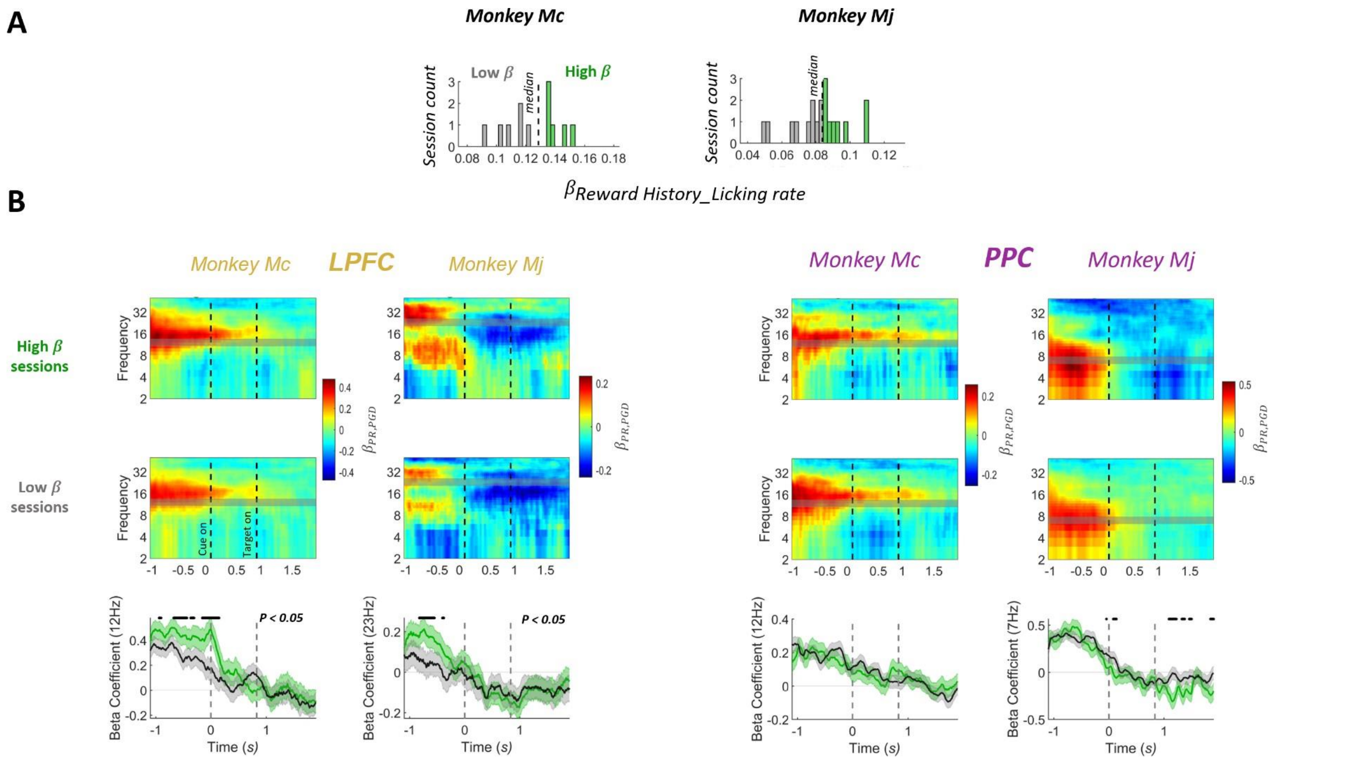

Fig. S5. Alternative session-level analysis of the relation between PGD and behavior (A)** For each session, we extracted a GLM coefficient measuring the sensitivity to the prior reward in the monkeys' pre-cue licking behavior ($b_{Reward\ History\_Licking\ rate}$) and performed a median split of sessions with high versus low sensitivity. **(B) Top two rows: GLM coefficients measuring the PGD sensitivity to prior reward on sessions with high versus low behavioral sensitivity.** All conventions as in **Fig. 5C. Bottom row: GLM coefficients at each animal's peak TW frequency** (gray band in the panels above). Shaded error bars represent SEM. Stars indicate time points in which the PGD sensitivity is higher in sessions with high versus low sensitivity ($p <0.05$, two sided t-test). Significant differences are found in the LPFC but not PPC for both monkeys, confirming the main analysis in **Fig. 5.
