# Supplementary material for: Beta traveling waves in monkey frontal and parietal areas encode recent reward history
Source: Nat Commun. 2023 Sep 5;14:5428. doi: 10.1038/s41467-023-41125-9 (PMC10480436; doi:10.1038/s41467-023-41125-9)
Supplement: Supplementary file 3 — Description of Additional Supplementary Files [file 41467_2023_41125_MOESM3_ESM.pdf]

Description of Additional Supplementary Files

Supplementary Movie 1: Example video showing one representative TW. Data show an example trial (recording session 3, trial 175) from monkey Mj's array PPC. Animation includes the phase values of an 11.8-Hz oscillation and the illustration of fitting a traveling wave on the spatial phase distribution. The instantaneous properties of the wave, including speed and direction, are also illustrated. The single red arrow represents the mean direction across electrodes.
